# Transport current and magnetization of Bi-2212 wires above liquid Helium temperature for cryogen-free applications

**DOI:** 10.1038/s41598-021-91222-2

**Published:** 2021-06-03

**Authors:** A. Leveratto, A. Angrisani Armenio, A. Traverso, G. De Marzi, G. Celentano, A. Malagoli

**Affiliations:** 1 CNR-SPIN, Corso Perrone 24, 16152 Genoa, Italy; 2 ENEA, Superconductivity Laboratory, Frascati Research Centre, Via E. Fermi, 45, 00044 Frascati, Italy; 3 Physics Department, Università di Genova, Via Dodecaneso 33, 16146 Genoa, Italy

**Keywords:** Superconducting properties and materials, Magnetic properties and materials

## Abstract

Since the discovery of high temperature superconductors, a possible cryogen-free scenario has always been wished. Nowadays, liquid Helium is running out, and it is likely that the cooling by will be a large part of the costs of any superconducting system. Bi-2212 wires at temperature higher than 4.2 K still show a very high irreversibility field and thus a deep investigation of their properties in such a range of temperature is very useful in order to assess the applicability in high field cryogen-free magnets. Here electrical transport and magnetic properties characterization at variable temperature and magnetic field on our “GDG—processed” wires are reported together with a well-described original approach to calculate the irreversibility field *H*_irr_. This study is devoted to provide reference data on the behaviour of the only isotropic wire for high field application with an eye to the performances at temperatures above 4.2 K.

## Introduction

Helium shortage is now a serious problem for the Research Centres and Universities as well as for industry. The Helium shortage will continue through thick and thin into 2021 and beyond^1,2^ and the costs for cooling NMR and MRI superconducting will rise. It is expected indeed a double-digit percent increase. Such an aggressive price increase is not likely to free up much gas and, as a consequence, in situations where liquid Helium substituting or recycling is possible, the tightened supply and higher costs are prompting efforts in both academia and industry to convert to dry cryostats, or closed-cycle refrigerant units, which eliminate the need to replenishing.

Over the past 20 years, advances in technology and industrial development led to the commercial availability of cryocoolers and cryogenic-free systems capable of operating easily at temperatures below 20 K. Typically, these systems consist of two stages: the first cooling down to about 60 K, while the second can reach below 10 K. With the use, in these systems, of a third stage employing rare earths, the possibility of operating at 4.2 K, and therefore in principle to replace liquid Helium, has become reality^3^. The additional complications and costs brought by this third stage have to be taken into account to evaluate the real convenience. However, other parameters and technical aspects have an impact too, in some cases even greater, on the costs and reliability of a cryogen-free system, strictly depending on the working temperature. A commercial system with an input power of about 2 kW can provide a cooling power of 20 W at 20 K, 6 W at 10 K and only 1 W at 4.2 K^3^. The majority of the metals used to build parts of the cryostat and the magnets (or conductor used for) to be cooled, such as for example Copper and Silver, show an increment of the thermal capacity of a factor 20 and 100 passing from 4.2 K to respectively 10 K and 20 K^4^. Similar behaviour is also found with regard to thermal conductivity, which shows an enhancement of a factor 2 at 10 K and 3 at 20 K^5^. These parameters are preferred to be as high as possible to have high efficiency and thermal stability in both the cooling system and in the superconducting magnets. Finally, at temperature below 20 K, the system is more demanding in terms of thermal insulation, which entails a more complicated and expensive construction technology. Therefore, it is clear that being able to operate devices, such as superconducting magnets, at temperatures around 20 K or at least above liquid Helium boiling temperature represents a great advantage. This makes very important and useful for magnet manufacturers to have access to a thorough and exhaustive properties characterization of the technological superconductors in this temperature range. If we consider the applications at medium–high magnetic fields, i.e. above 10 T such as in nuclear fusion or NMR, we have to refer to HTS rather than LTS superconductors.

Bi-2212 wires are the only HTS superconductor having a wire shape sought-after by the magnet builders for its isotropy. The wire performances are generally directly connected with the powder quality, and it depends on its composition, phase assemblage, particle size and distribution, the content of carbon and impurity elements. Until 2014 Nexans in Germany was the only Bi-2212 powder supplier, and the great majority of the studies and works on Bi-2212 wires published, even recently, were based on such precursor powders. Few data are available in literature about the properties at temperatures higher than 4.2 K. In ref. ^6^ the Kramer field *H*_K_, an approximation of the irreversibility field *H*_irr_, is reported for a Bi-2212 round wire being higher than 30 T at around 10 K, and *H*_K _≈ 9 T around 20 K.

Engi-Mat made significant progress in manufacturing Bi-2212 powder, which recently demonstrated to have excellent performances, the highest in terms of critical current density *J*_C_ of the wire at 4.2 K^7^.

In this work, we fabricated Bi-2212 wires using the Engi-Mat co. (previously operated as nGimat Co./LLC) Bi-2212 powders and processed by our innovative GDG—P.I.T. (Groove Die Groove—Powder In Tube) method described in^8^, which is a possible alternative to the Over Pressure OP method^9^, at least for those processes in which this last is not easily applicable^10^. The OP process has the aim to enhance the powder density inside the wire. Its great success in terms of *J*_*C*_, however, is offset by the complexity and costs, especially for large magnets. The GDG process, whose aim is again to enhance the powder density through a proper alternation of drawing and groove rolling, did not lead so far to so high *J*_C_, but high enough for applications and its strength is the scalability on industrial level.

Recently our group, ref. ^11^, reported about the behaviour of the transport properties as a function of temperature of a GDG processed wire with Nexans powders, useful now for a possible comparison.

In this paper we report on the superconducting properties at different temperatures measured on an innovative wire with the best commercially available powder. In particular, we will show critical current measurements up to 7 T in a range of temperature between 4.2 and 20 K and magnetic measurements performed by Vibrating Sample Magnetometer (VSM) up to 8 T between 8 K and about 80 K. An analysis of the difference, for what concern the irreversibility field, between the two main powder precursors suppliers for Bi-2212 wire will be discussed. As a further result, we provide an evaluation of the ranges of applicability of these superconducting wires in terms of temperature and magnetic field that can be a base for the magnet projects development.

The originality of this work lies not only on the fact that the literature is very poor about such kind of characterization referred to Bi-2212 wires in general, but also and mostly on the Bi-2212 wires themselves analysed here.

## Results

### Critical current measurements

**Figure 1:**
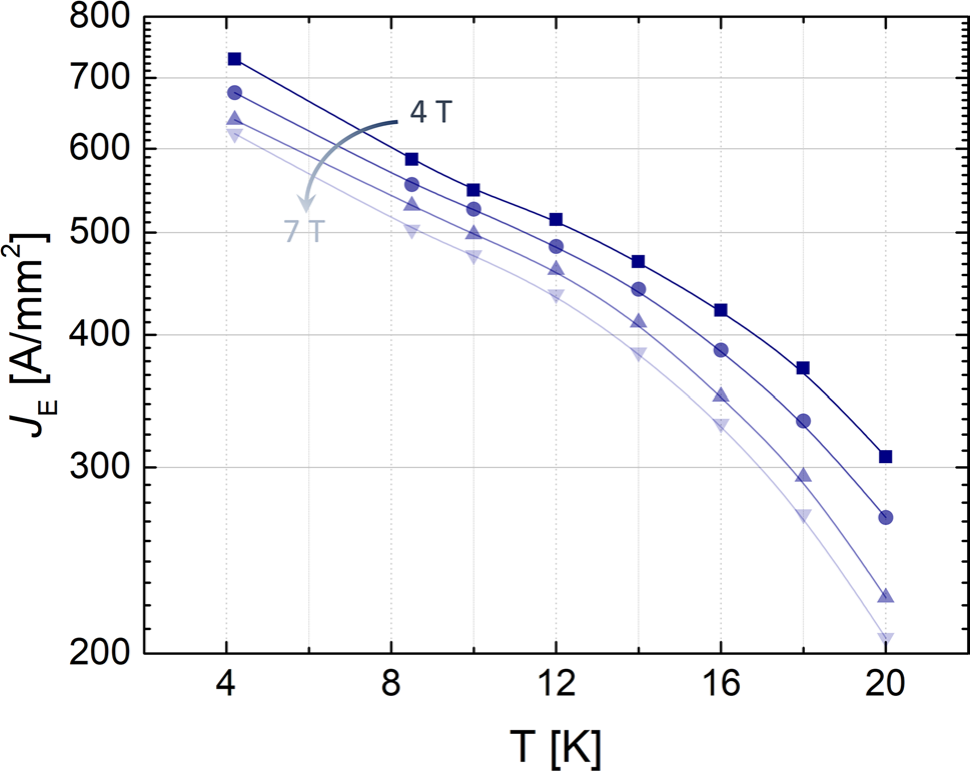
*J*_E_ as a function of the temperature at different magnetic fields.

**Figure 2:**
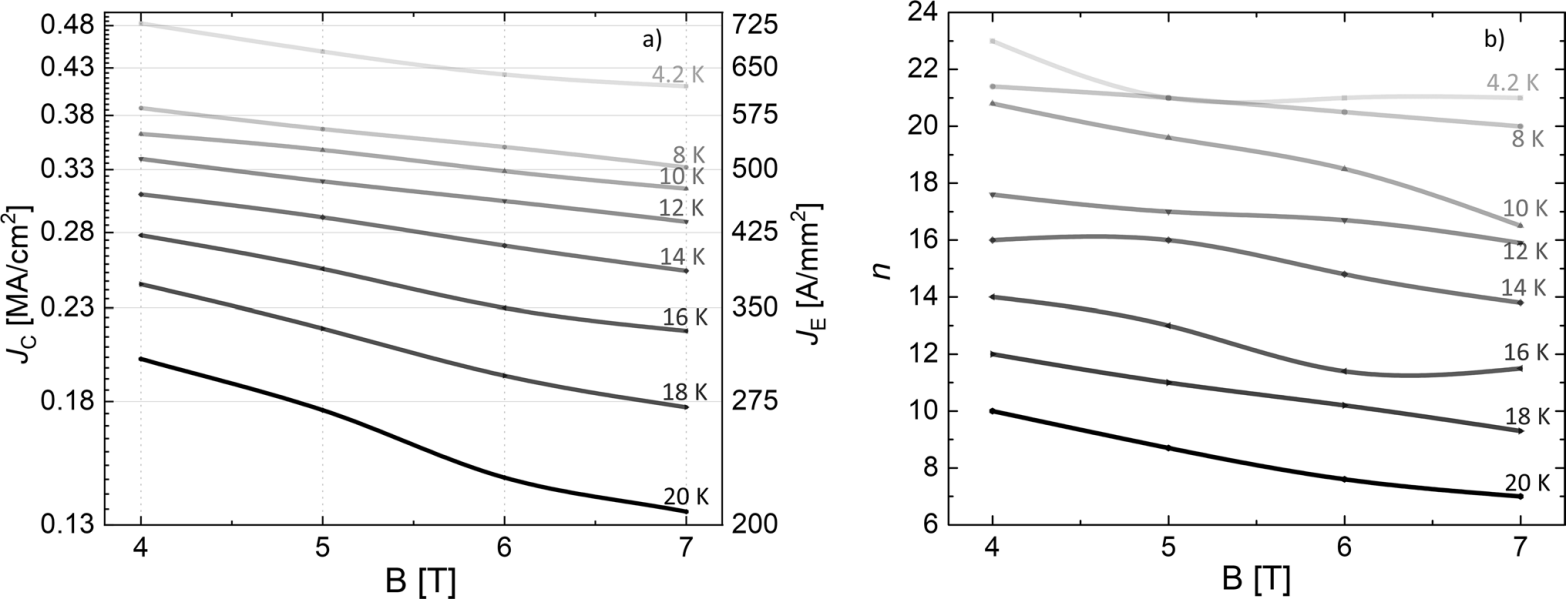
(**a**) *J*_C_ and *J*_E_ and (**b**) *n* values from V–I curves ($V = v_{o} I^{n}$) as a function of the applied magnetic field at different temperatures.

Figure 1 shows the measured engineering critical current density *J*_﻿E_ as a function of the temperature for different applied magnetic fields. In Fig. 2a) their behaviour with respect to the applied field is reported for different temperatures. From these graphs it can be seen that not only the critical current behaviour is very slightly field dependent in the measured field range, as indeed expected for Bi-2212, but it is the same for temperatures up to 12–14 K. For higher temperatures, a faster decrease of the transport properties is evident. However, at 20 K the samples still maintain a remarkable *J*_E_ > 200 A/mm^2^ up to 7 T. Figure 2b reports the n-values obtained from the V-I curves.

### Vibrating sample magnetometer measurements

**Figure 3:**
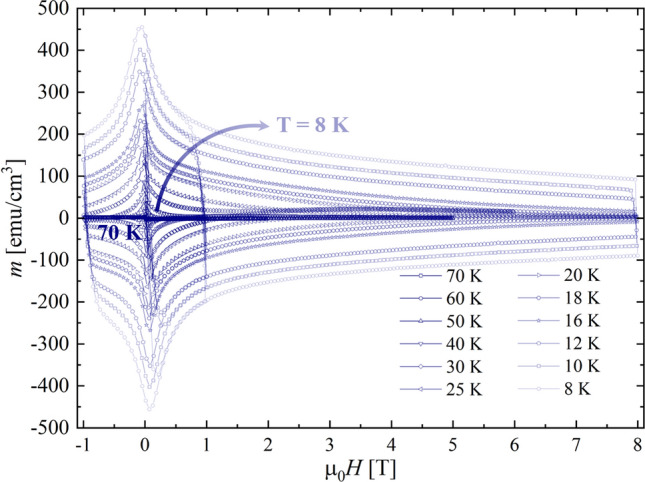
Magnetisation as a function of the applied magnetic field, up to 8 T, recorded at 70, 60, 50, 40, 30, 25, 20, 16, 12, 10 and 8 K.

The magnetization hysteresis loops as a function of the applied magnetic field (*M*-*H*), recorded at different temperatures, for a small piece length of about 0.515 cm cut from the same sample used for *J*_C_ measurements, with the applied magnetic field perpendicular to the wire axis, are reported in Fig. 3. The irreversibility field, *H*_irr_, can be directly obtained from the *M*-*H* curves, as the field at which the cycles close, only for *T* > 27 K, where *H*_irr_ is lower than the maximum experimentally achievable magnetic field (8 T). For lower temperatures *H*_irr_ can be estimated considering the functional expression of the macroscopic pinning force density *F*_P_ = *J*_C_ × μ_0_*H*.

From the hysteresis loop, applying the Bean critical state model^12^, the pinning force can be ultimately derived.

**Figure 4:**
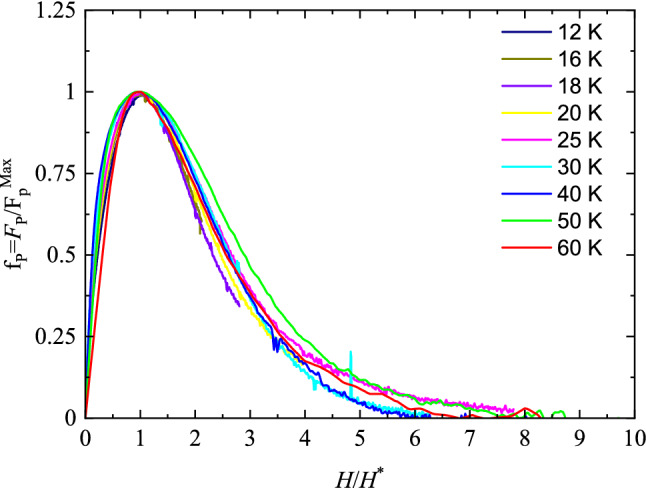
Reduced pinning force density *f*_p_ = *F*_p_/*F*_pmax_ as a function of the reduced field *H*/H*, at 60, 50, 40, 30, 25, 20, 16, 12 K.

Figure 4 shows the reduced pinning force density *F*_P_/*F*_Pmax_ (*F*_Pmax_ is the maximum value of the pinning force density) curves plotted as a function of the reduced field *H*/H* (H* is the magnetic field corresponding to *F*_Pmax_) within the temperature range 12–60 K in which it is possible to evaluate *F*_Pmax_, whereas at 8 K and 10 K, the maximum of pinning was not achieved within the investigated magnetic field (0–8 T) hence those curves are not shown. It is evident, from Fig. 4, that all the curves do not collapse on a single one when evaluated at different temperatures. The lack of the scaling behaviour of the pinning force in such an extended temperature range was often reported for Bi-2212 wires and tape and ascribed to the progressive change in pinning character from 2 to 3D, as *T* decreases^13,14^.

Following the Kramer model, the dependence of pinning force normalized to *F*_Pmax_ at a given temperature can be expressed as^15^:

1 $$f_{P} \left( {H/H_{irr} } \right) = \frac{{F_{P} }}{{F_{Pmax} }} = a \left( {\frac{H}{{H_{irr} }}} \right)^{p} \left( {1 - \frac{H}{{H_{irr} }}} \right)^{q} ,$$

where the magnetic field was in the original work normalized to *H*_c2_ value at the temperature of interest but, for HTS, Eq. (1) holds by normalizing to *H*_irr_. The *a* value is strictly related to *p* and *q* parameters throughout the relationship $a = { }\left( {\frac{p}{p + q}} \right)^{ - p} \left( {\frac{q}{p + q}} \right)^{ - q} .$

Since for the investigated sample the pinning forces scaling cannot be assumed, this implies that the *p* and *q* parameters will depend upon temperature.

The temperature behaviour for *T* > 27 K of both *p* = *p*(*T*) and *q* = *q*(*T*) is obtained by fitting the experimental *f*_P_(H) with Eq. (1), assuming the *H*_irr_ values determined from *M*-*H* loops, and *p* and *q* as fitting parameters. The *q* parameter reveals a smooth decreasing trend with increasing temperature within the range 27 K < *T* < 40 K, whereas *p* exhibits a rather *T*-independent behaviour. As example, the obtained values at 27 K are *p* = (0.64 ± 0.01) and *q* = (3.89 ± 0.01)^16,17^.

At this point, a comment on the meaning of the obtained parameters is necessary. As demonstrated by Dew-Hughes, *p* and *q* exponents in Eq. (1) are related to the geometry of defects and their nature (superconducting or normal) providing a powerful indication of the main pinning mechanisms active in a sample under investigation. However, the present values are rather inconsistent with those predicted by Dew-Hughes models, thus a dominant pinning mechanism cannot be unambiguously identified. This is not a unique behaviour of the wires investigated in this work but, on the contrary, the obtained *p* and *q* values well agree with those previously reported for similar systems, thus a quite general behaviour of Bi-2212 wires and tapes. Nevertheless, the pinning force density of Bi-2212 wires are often discussed considering the characteristic dependence of grain boundary pinning with *p* = 0.5 and *q* = 2^18^. The higher values reported for Bi-2212 samples can be ascribed to the microstructural nature with randomly distributed *c*-axis orientation in wire radial direction. Due to anisotropic properties of *H*_irr_ in Bi-2212, the resulting macroscopic behaviour is determined by the co-existence of grains with different *H*_irr_ and then dissipative state. In this scenario, a smoother behaviour (and thus higher *q* values) in high field condition approaching *H*_irr_ is justified^14^.

**Figure 5:**
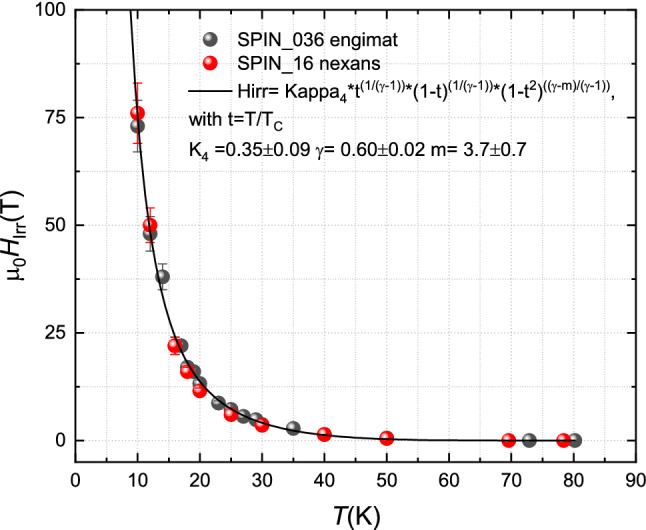
Irreversibility field as a function of temperature of both SPIN_036engimat (black circles) and SPIN_016nexans (red cycles). The expression used for calculation is in agreement with the reference reported. The black line represents the fit of SPIN_036engimat *H*_irr_(*T*) with the equation in^16^. The values of fitting parameters are also shown from which a good matching with *H*_irr_(*T*) curves obtained in^16^ can be inferred.

Coming back to the evaluation of *H*_irr_, for *T* < 27 K, the values of *H*_irr_ are estimated by fitting the experimental curves with the *f*_P_(*H*) functional dependence (Eq. 1) using *p* and *H*_irr_ as fitting parameters. The fitting procedure was performed keeping the *q* parameter at either *q* = 3.89 (the value at 27 K) or higher values *q* = 4, 4.25, 4.5 up to *q* = 5 (extrapolation of the *q*(*T*) linear dependence for *T* > 27 K in the low *T* limit). The final value associated to *H*_irr_ at any given *T* is then calculated as the average of *H*_irr_ obtained by the full set of fitting processes with different *q*. The as calculated *H*_irr_ values are plotted in Fig. 5 and reported in Table 1, with errors provided by the root mean square values.

**Table 1:** Irreversibility field, *H*_irr_, values of the two investigated Bi-2212 wires SPIN_016nexans and SPIN_036engimat. High temperature values are obtained by isothermal *M*-*H* hysteresis loops, whereas for *T* < 25 K (SPIN_016nexans) and 27 K (SPIN_036engimat), values are associated to the average value obtained by fitting with Eq. 1) (see text for more details) assuming different *q*. Data are also plotted in Fig. 5.

T (K)	*H*_irr_ (T)	*H*_irr_ (T)
	SPIN_016nexans	SPIN_036engimat
8	–	127 ± 11
10	76 ± 5	73 ± 5
12	50 ± 4	48 ± 4
14		38 ± 3
16	22 ± 2	22 ± 2
17		22 ± 1
18	16 ± 1	17 ± 1
19		16 ± 1
20	11.5 ± 0.7	13.2 ± 0.8
23	–	8.7 ± 0.5
25	6.0	7.2 ± 0.5
27	–	5.60
30	3.52	4.89
35		3.70
40	1.34	2.79
50	0.42	1.40
60	0.18	0.150
69.6	0.002	–
70	–	0.08
72.9	–	0.002
78.4	0	–
82	–	0

As a further contribution to the work, we try to compare the properties of these wires prepared with Engi-Mat powders with a very similar square wire realized with Bi-2212 powders produced by Nexans (batch n. 87), broadly used until 2017. This wire—here labelled SPIN_016nexans and thoroughly described in^8,11^—was also processed via GDG method and had the same final filament size. The big difference lies in the variation of about a factor 2 in the critical current density *J*_C_SPIN_016nexans_(4.2 K, 5 T) = 0.2 MA/cm^2^ and *J*_C_SPIN_036engimat_(4.2 K, 5 T) = 0.45 MA/cm^2^. A similar difference in *J*_*C*_ was reported also for the OP processed wires realized respectively with Nexans and Engi-Mat powders^7^. Adopting the same procedure described for SPIN_036engimat, *H*_irr_ has been calculated in the whole temperature range. The as obtained values are plotted in Fig. 5 and listed in Table 1 for an easier comparison with sample SPIN_036engimat.

As can be seen, in spite of the significantly different *J*_C_ performances recorded in the two Bi-2212 wires, their *H*_irr_(*T*) curves exhibit very similar temperature behaviour and similar values, in the limit of the experimental uncertainties, in the whole temperature range. In Fig. 5, the curve obtained by fitting the SPIN_036engimat *H*_irr_(*T*) with the predicted *T*-dependence for Bi-2212 wires is also plotted. A satisfactory fit can be obtained with fitting parameter values in good agreement with previously reported results for *H*_irr_ curves measured on Bi-2212 wires by dc transport methods^16^. This supports the validity of the proposed procedure for *H*_irr_ calculation.

The similarity of the obtained *H*_irr_(*T*) curves can be explained considering that both wires exhibit similar vortex pinning mechanisms. Hence, as already proposed for Bi-2212 wires manufactured with different precursors, the origin of the discrepancy in *J*_C_(*H*) behaviours must be more likely ascribed to microstructural issues such as higher density and better grain connectivity rather than in improved pinning mechanisms^7^.

## Discussion

The aim of this paper is to provide as comprehensive a characterization as possible of the transport properties above 4.2 K of one of the most attractive technological superconductors. The work was done on a Bi-2212 wire that combines an innovative and scalable fabrication process (GDG) with the best state-of-the-art commercial powders (Engi-Mat): a wire that therefore can really generate the interest of the magnet manufacturers. However, the considerations resulting from this study can be applied as well to the OP processed wires; indeed, in the OP process it has been shown^9^ how the Bi-2212 density is enhanced but none of the other properties, such as pinning, has been modified with the result to increase the absolute value of *J*_C_ even by a factor of 2–3, while maintaining its behaviour the same with respect to the magnetic field. These aspects make such a characterization particularly useful, above all because it gives an indication of the real possibilities of building devices that can work without the need for liquid Helium.

**Figure 6:**
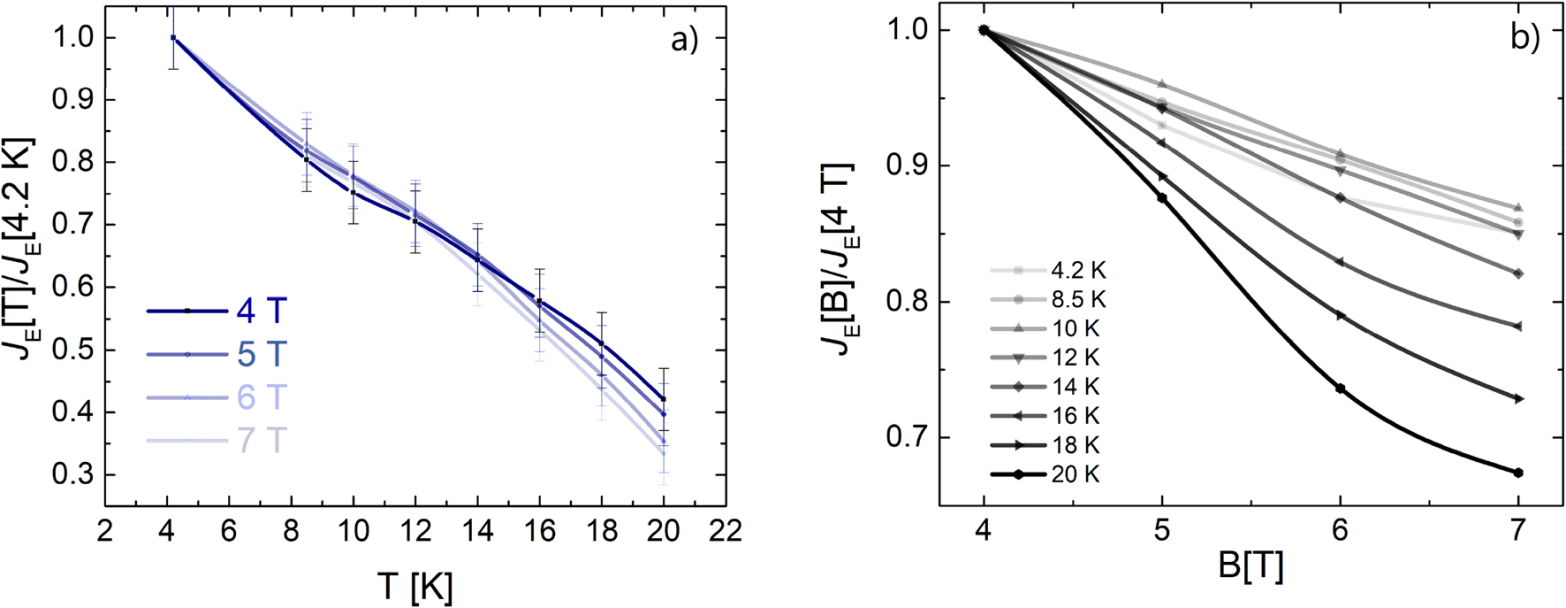
(**a**) Critical current density behaviour at different applied magnetic fields as a function of temperature; (**b**) critical current density behaviour at different temperature as a function of applied magnetic field.

For a better reading of the results, the engineering critical currents versus temperature for each field normalized to *J*_E_(4.2 K) and versus magnetic field for each temperature normalized to *J*_E_(4 T) have been reported respectively in Fig. 6a,b. We can observe the same (within the 5% of error) slope of the normalized *J*_E_ up to temperatures of about 14 K. For higher temperatures, a degradation increasing with the magnetic field is evident reaching the maximum decrease of about 30% at 20 K, 7 T. This can be explainable considering that at this temperature we are approaching the irreversibility magnetic field.

**Figure 7:**
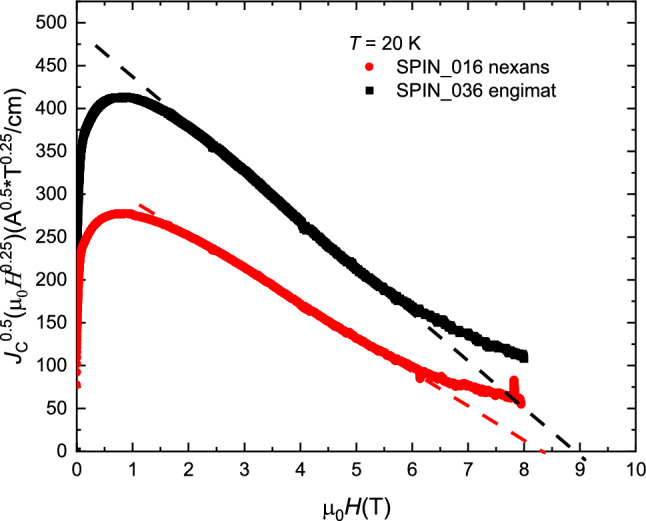
Fit of the linear part of the Kramer plot where is defined the H_K_ as the intercept of the applied field axis.

From our measurements of *H*_irr_ shown in Fig. 5 we obtained a value of about 13 T at 20 K. Looking at the few works reported in literature we can find only an estimation of *H*_irr_, the so-called *H*_K_, obtained by fitting the linear sections of Kramer plot *J*_C_^0.5^*B*^0.25^ *vs B* and considering the linear extrapolations to the field axis. In a recent work^7,19^, values of *H*_K_ of about 9 T at 20 K were reported for wires fabricated with the same powders used in the present work. Also in our GDG samples, if we look at the evaluation of *H*_K_ shown in Fig. 7, we see similar values and only a slight difference between the two wires perfectly in agreement with^19^. However, as described for example in^20^, the Kramer model works only for a few types of high-field superconductors, such as Nb_3_Sn, and has very limited success in representing other materials, such as HTS. In this work, following the proposed approach, *H*_irr_ is more consistently estimated in the whole temperature range showing that *H*_K_ largely underestimates the field range of applicability of Bi-2212 wires. For instance, a first result coming out from this approach is that at 20 K an irreversibility field of 13.2 ± 0.8 T is obtained instead of 9 T from Kramer fit, which is more consistent with the far from negligible *J*_E_ = 200 A/mm^2^ measured at 7 T.

As also our work confirms, the vortex pinning seems not playing any role in the improvement of *J*_C_ performances of Engi-Mat based wires, at least up to about 18 K. There might be some differences in pinning properties at temperature higher than 20 K which might be interesting to investigate further. However, up to now, regarding the better Engi-Mat performances at low temperatures, the most accredited hypothesis concerns the better connectivity, bi-axial texturing and grain boundaries cleanliness obtained with these new powders^19^. These findings indicate that there is still room for improvements by acting on the pinning mechanisms which could provide a strengthening of the magnetic field behavior and could extend the magnetic field range of applicability of Bi-2212 wires.

Summarizing the results, looking at 20 K, the temperature where we can get the maximum benefit from a cryogenic system in terms of available cooling capacity and thermal insulation, our GDG wires show *J*_E_ in excess of 200 A/mm^2^ at 7 T. The relatively low *H*_irr_ (13.2 ± 0.8 T) and the steep *T*-decrease of *J*_C_ close to this temperature strongly limit the applicability of these Bi-2212 wires at low-field conditions, e.g. medical applications such as magnets for hadron therapy.

The situation is different at 10 K. Here, we have a *J*_E_ of about 500 A/mm^2^ at 7 T and the *H*_irr_ is about 73 ± 5 T. At this temperature, due to the reported robust in-field behaviour of *J*_E_, high-field applications can be reasonably envisaged. Moreover, a good thermal stability can be inferred by the measured *J*_E_(T, H) behaviour. Thinking about a possible device, an operating current as high as 80% of *I*_C_ can be sustained at 10 K, 7 T with a temperature margin of about 4 K (as from Fig. 2). This aspect is important for the magnet builders because it can prevent issues due to possible temperature instability in a cryogen-free system. Moreover, operating with a cryocooler at 10 K still has some advantages with respect to 4.2 K in terms of cooling power, thermal capacity, and thermal conductivity, all parameters that determine high efficiency and thermal stability in both the cooling system and in the superconducting magnets.

## Conclusions

In a scenario that increasingly sees the use of cryogen-free systems instead of liquid Helium, the Bi-2212 can further strengthen its role as an attractive conductor for applications. This work shows a rather complete characterization of the conductor at temperatures above 4.2 K, being able, at the same time, to fill a gap in the literature on this superconductor. From the described results we can draw the following conclusions:

- Bi-2212 wire has been shown to be suitable for high magnetic field applications around 10 K, having good stability of *J*_C_—i.e. a non-abrupt decrease in magnetic field in the range 8–12 K—and an irreversibility field at 10 K higher than 70 T. These properties open a wide window of applicability in the temperature—field diagram. In fact, our original wires processed at 1 bar show at 10 K a *J*_E_ = 500 A/mm^2^ at 7 T promising to be in line with the application requirements also at high field; however, the absolute values can even rise if we think of the wires processed at 50/100 bar (OP).
- The two main Bi-2212 powders used up to now to fabricate P.I.T. wires—Nexans and Engi-Mat—led to very different transport properties of the conductors, being *J*_C_ of Engi-Mat conductors about twice higher than Nexans ones. Our analysis seems to clarify that there are no different pinning properties below 20 K and, therefore, the reasons for their different effect have to be found in other aspects such as connectivity and grain boundaries cleanliness.
- An original and consistent approaching method has been proposed to calculate the irreversibility field which overcomes the approximation brought by the Kramer plot. We think that such a method might be used as a reference for future works and analysis.

## Methods

### Sample preparation

**Figure 8:**
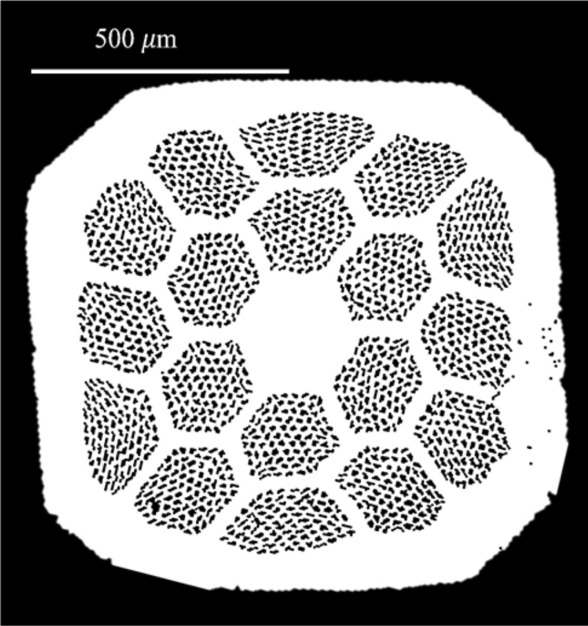
Cross section of SPIN_036engimat.

Bi-2212 multifilamentary wires were prepared through the P.I.T. technique adopting our new concept for the densification of powders before the partial melt process (PMP), the so-called GDG process^8^: the wire is cold deformed through groove-rolling steps properly alternating with drawing steps. The Bi-2212 powder used here was produced by Engi-Mat (batch n. KZA-87-67H) whose composition was Bi_2.16_Sr_1.93_Ca_0.89_Cu_2.02_O_x_. A pure Ag tube with outer (OD) and inner (ID) diameters of 15 and 11 mm respectively was filled with the powder in O_2_-atmosphere and drawn. The obtained monofilamentary wire was cut into 85 pieces and restacked in a second 13/11.5 mm (OD/ID) pure Ag tube. From here on, the deformation process followed the GDG method. The resulting wire was hexagonally shaped, cut in 18 pieces + 1 (pure silver wire prepared ad hoc and placed in the centre) and restacked in a 15.2/12.7 mm (OD/ID) Ag tube. Finally, a 1530 filaments (85 × 18) square wire with a size of about 1 × 1 mm^2^ is obtained with a superconducting filling factor of about 15%. The average filament diameter was 12 µm. 12 cm long samples were heat treated in 1 bar flowing O_2_ in a three-zone tubular furnace with a homogeneity of ± 0.5 °C in 30 cm using the standard HT schedule^21^. Figure 8 shows the transversal cross sections of the green wire (before the heat treatment) labelled as SPIN_036engimat.

### Sample characterization

The transport critical currents as a function of variable magnetic field and temperature were measured by means of a built-in-house system, previously described in^22^ and using the 1 µV/cm criterion. The temperature was ranged between 4.2 and 20 K, while the magnetic field between 0 and 7 T. The magnetic properties were characterized by means of an Oxford vibrating sample magnetometer (VSM) equipment provided with an 8 T superconducting magnet. Magnetization cycles, up to 8 T, as a function of the applied magnetic field at several temperatures were recorded with a field ramp rate of 0.3 T min^−1^. The superconductor critical state parameter was derived assuming the Bean model.
